# Activity-Based Anorexia Induces Browning of Adipose Tissue Independent of Hypothalamic AMPK

**DOI:** 10.3389/fendo.2021.669980

**Published:** 2021-06-02

**Authors:** Angela Fraga, Eva Rial-Pensado, Rubén Nogueiras, Johan Fernø, Carlos Diéguez, Emilio Gutierrez, Miguel López

**Affiliations:** ^1^ Department of Physiology, Center for Research in Molecular Medicine and Chronic Diseases (CiMUS), University of Santiago de Compostela-Instituto de Investigación Sanitaria, Santiago de Compostela, Spain; ^2^ CIBER Fisiopatología de la Obesidad y Nutrición (CIBERobn), Santiago de Compostela, Spain; ^3^ Department of Clinical Psychology and Psychobiology, School of Psychology, University of Santiago de Compostela-Instituto de Investigación Sanitaria, Santiago de Compostela, Spain; ^4^ Hormone Laboratory, Haukeland University Hospital, Bergen, Norway; ^5^ Unidad Venres Clínicos, School of Psychology, Universidad of Santiago de Compostela, Santiago de Compostela, Spain

**Keywords:** activity-based anorexia, temperature, cachexia, brown adipose tissue, white adipose tissue, hypothalamus, AMPK, ER stress

## Abstract

Anorexia nervosa (AN) is an eating disorder leading to malnutrition and, ultimately, to energy wasting and cachexia. Rodents develop activity-based anorexia (ABA) when simultaneously exposed to a restricted feeding schedule and allowed free access to running wheels. These conditions lead to a life-threatening reduction in body weight, resembling AN in human patients. Here, we investigate the effect of ABA on whole body energy homeostasis at different housing temperatures. Our data show that ABA rats develop hyperactivity and hypophagia, which account for a massive body weight loss and muscle cachexia, as well as reduced uncoupling protein 1 (UCP1) expression in brown adipose tissue (BAT), but increased browning of white adipose tissue (WAT). Increased housing temperature reverses not only the hyperactivity and weight loss of animals exposed to the ABA model, but also hypothermia and loss of body and muscle mass. Notably, despite the major metabolic impact of ABA, none of the changes observed are associated to changes in key hypothalamic pathways modulating energy metabolism, such as AMP-activated protein kinase (AMPK) or endoplasmic reticulum (ER) stress. Overall, this evidence indicates that although temperature control may account for an improvement of AN, key hypothalamic pathways regulating thermogenesis, such as AMPK and ER stress, are unlikely involved in later stages of the pathophysiology of this devastating disease.

## Introduction

Anorexia nervosa (AN) is an eating disorder characterized by decreased food intake, severe weight loss and hyperactivity (1, 2). Due to chronic underfeeding, patients with AN present neuroendocrine changes, in an attempt to adapt to malnutrition, which in many cases are not completely reversed even with the recovery of body weight (3); this leads to several medical complications (4, 5).

Activity-Based Anorexia (ABA) is considered the best analogue animal model for AN (6), which is obtained by providing availability of food to rats 1-2 h/day and free access to a running wheel (7). Under these circumstances, rats develop an excessive running and reduced meal efficiency, eliciting massive weight loss and hypothermia, both mimicking the principal signs of AN disorder in humans. Notably, ABA also reproduces the metabolic and endocrine abnormalities observed in humans (8). AN-associated hyperactivity has been proposed as an adaptative behavioral response to compensate for hypothermia (9). Previous research has shown that exposure to a high ambient temperature (AT) prevents and reverses the hyperactivity and improves feeding patterns, allowing body weight recovery in both male and female rats under ABA conditions (10–15). These beneficial effects of temperature have been also found in the semi-starvation induced hyperactivity model (SIH) (16).

Due to the ability of the ABA model to reproduce many of the symptoms of the AN disorder in humans, as well as the identification of several genes involved in food intake regulation and energy balance as potential pathways that contribute to the etiology and maintenance of AN (17, 18), it would be interesting to examine the effect of high AT on energy sensors potentially involved in AN, as well as the possible clinical implications on the treatment of AN in humans. Here, we focused on AMP-activated protein kinase (AMPK) and endoplasmic reticulum (ER) stress, well-known mechanisms regulating both sides of the energy metabolism, namely feeding and thermogenesis (19–26).

## Materials and Method

### Animals

Male Sprague-Dawley rats (130-190 g) were acquired from the Animalario General USC, (Santiago de Compostela, Spain). They were kept with food and water *ad libitum* on a 12-hr light-dark cycle (LD, lights on from 08:00 to 20:00 hours). Ambient temperature set at 21 ± 1°C. The Ethics Committee on the use and care of animals of Santiago de Compostela University approved all described procedures (project license 15004/17/002). All experiments were carried out in accordance with Royal Decree 53/2013 of February 1, Law 32/2007 of November 7, and European Communities Council Directive 2010/63/UE of September 22, on the protection of animals used for experimental and other scientific purposes.

### Running Wheels

Cages (48 x 31.5 x 47 cm) equipped with a Whatman-type activity wheel (1.12-m circumference 35.7 cm diameter, 10-cm-wide running surface of a 10-mm mesh bounded by clear Plexiglas and stainless-steel walls; *Panlab Harvard Apparatus;* Barcelona, Spain) were placed inside wooden incubators (60 × 60 × 60 cm) with polycarbonate roofs, provided with a 150 W heat wave lamp, connected to a thermostat and a probe positioned at the level of the animal, which allowed individual control of AT.

### ABA Procedure

One week prior to the start of the experiments body temperature and activity transmitters (*PTD 4000 E-Mitter*, *Respironics Mini Mitter Inc*; Bend, OR, US) were implanted under ketamine-xylazine anesthesia (50 mg/kg, intraperitoneal) and inserted in a subcutaneous pocket on the ventral surface created using blunt dissection. The rats were allowed seven days to recover. On the eighth day, rats were weighed and assigned to two weight matched groups: an active and restrictive-fed (AC) group and an inactive and restrictive-fed (IN) group. All the rats were transferred to running wheel cages, but only the rats assigned to the active condition had access to functional wheels. The rats assigned to the inactive condition remained with the activity wheel blocked during the whole experiment, avoiding any possibility of movement inside those devices. The ABA procedure started (day 0) with the removal of food at 12:30 h for restricted-fed groups. At the same time, the doors to the wheels were opened for the active group. From day 1 onward, all rats were given access to food according to a restricted feeding schedule from 11:00 to 12:30 h. The doors of the wheels were closed during this feeding period. Food intake was measured by weighing the food at the beginning and the end of every 1.5 h feeding period. Rats were also weighed daily at 10:30 h (as they were on day 0). This phase continued for each restricted-fed active rats until it reached a body weight loss criterion (BWLC) of 20% of their day 0 body weight. At this time, rats were assigned to one of two ambient temperature, 21°C or 32°C, as indicated in the two digits of the abbreviated group name (AC21 and AC32). These conditions were maintained until rats reached either the recovery criterion, which was defined as body weight on any particular day (day *n*) greater than the weight of the animal 4 days before, (day *n*-4), or the removal criterion which was defined as body weight under 75% of body weight on day 0 (7). The experiment was terminated after 15 days. The restricted-fed inactive rats were also assigned to two ambient temperature conditions, 21°C or 32°C (IN21 and IN32). For the rats maintained at 21°C the experiment lasted only six days (median of days that the restricted-fed active animals took to reach the BWLC). While rats assigned to 21°C AT remained in these conditions for three days more (median of days that de AC21 group took to reach the removal criterion), rats assigned to 32°C remained six days on experiment (median of days that the AC32 group took to reach the recovery criterion). At the end of the experiments rats were sacrificed by decapitation after the weighing routine; trunk blood, brain, brown adipose tissue (BAT), hind leg muscle and gonadal white adipose tissue (gWAT) were collected, frozen and stored at -80°C until assay.

### Blood Biochemistry

Trunk blood was collected into specific tubes (*BD Vacutainer*; Plymouth, UK) and centrifuged at 3,200x g for 15 min at 4°C to separate the serum. Then serum was stored at -80°C. Glucose free T3 and free T4 were measured using an automated chemistry analyzer (*ADVIA 2400 Chemistry System, Siemens Medical Solutions Inc;* Ann Arbor, MI US). Leptin, corticosterone (CORT), adrenaline and noradrenaline levels were measured using ELISA kits (*EZRL-83K; Linco Research;* St. Charles, Missouri, US, for leptin; *ab108821, Abcam*, Cambridge, UK, for CORT; *EIA-3175; DRG Instruments GmbH*, Marburg, Germany, for adrenaline and noradrenaline).

### Hypothalamic Dissection

The brain was placed in an adult rat brain matrix (*Kent-Scientific Corporation, #RBMA-300C*; Torrington, CT, US) with the hypothalamus upward and dissected as previously described (23, 24, 26–28).

### Western Blotting

BAT, gWAT and the hypothalamic nuclei (arcuate, ARC, and ventromedial, VMH) were homogenized in lysis buffer containing protease inhibitor cocktail tablets (*Roche Diagnostics*; Indianapolis, IN, US) and the protein concentration was determined using the Bradford method (*Protein assay dye concentrate, Bio-Rad Laboratories*; Hercules, CA, US). The protein lysates were subjected to SDS-PAGE and electro-transferred to polyvinylidene difluoride membranes (PVDF; *Millipore;* Billerica, MA, US) with a semidry blotter. Membranes were blocked in TBS/Tween with 3% of BSA (Bovine serum albumin, Sigma Aldrich, St. Louis, US) and probed with the following antibodies against: pAMPKα (Thr172), glucose-regulated protein 78 (GRP78; *Cell Signaling*; Danvers; MA, US), UCP1 (uncoupling protein 1; *Abcam*; Cambridge, UK), C/EBP Homologous Protein (CHOP; *SCBT*; Dallas, Texas, USA), α-tubulin and β-actin (*Sigma-Aldrich*; St. Louis, MO, US), as previously shown (23, 24, 26–29). Membranes were incubated with the corresponding secondary antibody: anti-rabbit, anti-mouse, or anti-goat (*DAKO*; Glostrup, Denmark). Detection of proteins was performed with Enhanced chemiluminescence (ECL) reagents (*Pierce ECL Western Blotting Substrate*, *Cultek*; Madrid, Spain) according to the manufacturer’s instructions, exposed to x-ray films (*Fujifilm*; Tokyo, Japan), developed and fixed under appropriate dark room conditions. Autoradiographic films were scanned and the bands signal was quantified by densitometry using *ImageJ-1.33 software* (NIH; Bethesda, MD, US), as shown (23, 24, 26–29). Values were expressed in relation to β-actin (hypothalamus) or α-tubulin (BAT). Representative images for all proteins are shown, all the bands for each picture always come from the same gel, although they may be spliced for clarity, as represented by vertical lines.

### Real-Time Quantitative RT-PCR

Real-time PCR (*TaqMan^®^; Applied Biosystems*; Foster City, CA, USA) was performed using specific primers and probes (**Supplementary Table 1**), as shown (27–30). Values were expressed in relation to hypoxanthine-guanine phosphoribosyl-transferase (*Hprt*) levels.

### Hematoxylin-Eosin Staining and UCP1 Immunohistochemistry

gWAT depots were fixed in 10% buffered formaldehyde and subsequently treated for histological study by dehydration (increasing alcohol concentrations), mounting in xylene and immersion in paraffin. The paraffin blocks were sliced into 3 mm sections that were processed, deparaffinized in xylene, rehydrated and rinsed in distilled water and then stained either for hematoxylin-eosin or UCP1 immunohistochemistry. For the hematoxylin-eosin processing, slices were first stained with hematoxylin for 5 min, washed and stained again with eosin for 1 min. For UCP1 immunohistochemistry, slices were incubated overnight with the primary antibody (UCP1; *Abcam*; Cambridge, UK), washed and incubated with the secondary antibody (*DAKO*; Glostrup, Denmark). Images were taken in an optical microscope with a digital camera *Olympus XC50* (*Olympus Corporation; Tokyo*, Japan) at 40X. Adipocyte area and UCP1 staining area were quantified using *ImageJ 1.33 software* (*NIH*, Bethesda, MD, US), as shown (25, 29, 31, 32).

### Statistical Analysis

Data are presented as mean ± SEM. When two groups were compared, statistical significance was determined by two-sided Student’s t-test; when more than groups were compared, statistical significance was determined by ANOVA followed by Bonferroni’s test. P < 0.05 was considered significant. Statistical analyses were performed using *SPSS 21.0 software* (*IBM*; Armonk, NY, US).

## Results

### Increased Housing Temperature Reverses the Effect of ABA on Energy Balance and Activity

**Figure 1:**
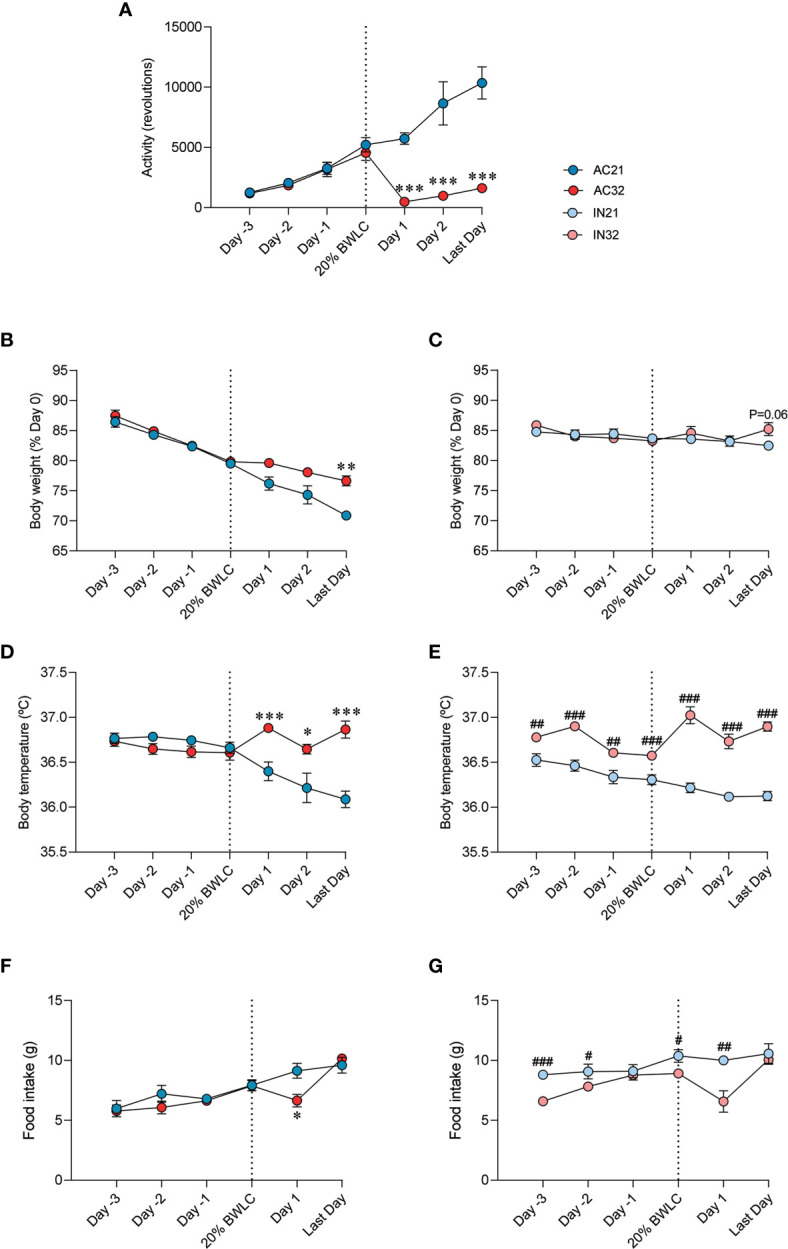
Effect of ABA and temperature on energy balance. **(A)** Activity (n = 9-12 rats/group) **(B, C)** Body weight (% of day 0) (n = 8-12 rats/group) **(D, E)** Body temperature (n = 7-10 rats/group) **(F, G)** Food intake (n = 8-12 rats/group) of active rats at 21°C and 32°C (AC21 and AC32) and inactive rats at 21°C and 32°C (IN21 and IN32) *P < 0.05, **P < 0.01, ***P < 0.001 *vs.* AC21; ^#^P < 0.05, ^##^P < 0.01, ^###^P < 0.001 *vs.* IN21. Data expressed as mean ± SEM. 20% BWLC, 20% body weight loss criterion.

ABA rats exposed at a housing temperature of 32°C (AC32) ran six-fold less than rats housed at 21°C (AC21). During Phase II running activity of AC32 rats did not reach the activity level shown on the last day of Phase I, (day they met the body weight loss criterion of 20%, 20%BWLC), despite being an average of 4 more days being subjected to standard ABA conditions (restricted feeding plus wheel access) (**Figure 1A**). Active rats lose weight during Phase II while inactive rats keep it stable. Besides, the increase in room temperature to 32°C allowed for less weight loss in active rats and slightly increased weight in inactive rats, when compared to their counterparts at 21°C (**Figures 1B, C**). Both active and inactive rats exhibited higher body temperature when they were maintained at 32°C, as (**Figures 1D, E**). Rats kept at lower temperature of 21°C initially ate more than rats at 32°C although no significant differences were detected on the final day (**Figures 1F, G**).

### Increased Housing Temperature Reverses the Effects of ABA on Circulating Parameters

Next, we evaluated the effect of housing temperature on circulating parameters in the ABA model (**Table 1**). We first focused on leptin levels, since this hormone has been shown to have a controversial role in this model of disease (16, 33–39). Resembling the clinical evidence (34, 36, 37), our data showed that active rats had significantly lower circulating leptin levels that inactive ones, which were elevated after exposure of the rats to 32°C (**Table 1**). No major changes were detected in glycaemia. Regarding CORT, active rats housed at 21°C displayed the highest circulating levels of this hormone (**Table 1**), similarly to AN patients and other preclinical models (40–44), indicating greater stress. Notably, when maintained at 32°C, active rats normalized their circulating CORT, reaching even lower concentration than the inactive groups (**Table 1**). Thyroid hormones (T4 and T3) play a major role in the modulation of temperature (45, 46), and we investigated how their circulating levels were affected in our setting. Inactive rats displayed the expected correlation between ambient temperature and thyroid status. Interestingly, that effect was not evident in active rats, which showed lower T4 and T3 when kept at 21°C as compared to 32°C (**Table 1**). Finally, active rats at 21°C also showed higher levels of noradrenaline, that were reduced when housed at 32°C (**Table 1**).

**Table 1:** Serum parameters in the experimental groups.

	Active 21°C	Active 32°C	Inactive 21°C	Inactive 32°C
Leptin (ng/mL)	0.20 ± 0.002!!!	0.24 ± 0.005***	0.28 ± 0.02	0.29 ± 0.02
Glucose (mg/dL)	120.33 ± 8.29	137.75 ± 3.28*	129.86 ± 5.82	140.00 ± 3.49
Corticosterone (ng/mL)	1466.87 ± 125.28!!!	273.05 ± 43.77***	412.98 ± 63.40	435.91 ± 35.57
T4 (ng/dL)	0.98 ± 0.05!!!	1.54 ± 0.07***	1.89 ± 0.07	1.71 ± 0.04^#^
T3 (pg/mL)	1.51 ± 0.09!!!	2.24 ± 0.05***	3.68 ± 0.05	3.07 ± 0.09^###^
Adrenaline (ng/mL)	2.46 ± 0.50	2.16 ± 0.19	4.18 ± 1.21	3.43 ± 0.77
Noradrenaline (ng/mL)	2.64 ± 0.32	1.75 ± 0.17*	3.54 ± 0.58	3.58 ± 0.58

n = 9-11 animals/group. *P < 0.05 and ***P < 0.001 vs. AC21. ^#^P < 0.05, and ^###^P < 0.001 vs. IN21. !!!P < 0.001 IN21 vs. AC21.

### Increased Housing Temperature Reverses the Effects of ABA on WAT and Skeletal Muscle

**Figure 2:**
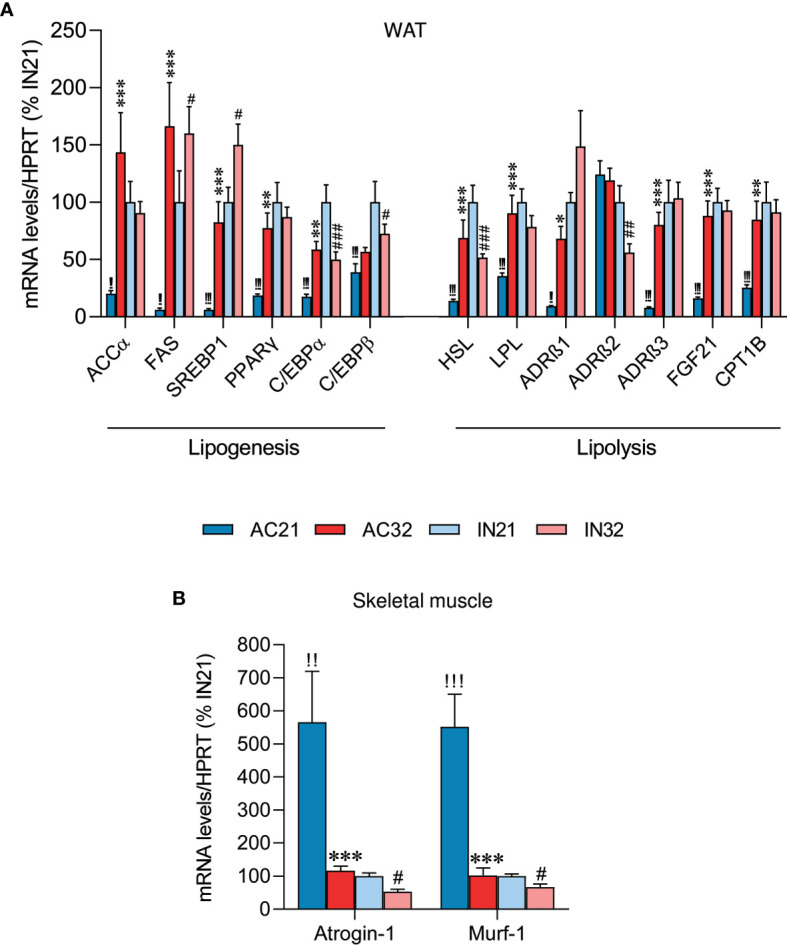
Effect of ABA and temperature on WAT and muscle. mRNA levels of **(A)** lipogenesis and lipolysis markers in the WAT (n = 8-11 rats/group) and **(B)** cachexia markers in skeletal muscle (n = 8-11 rats/group) of active rats at 21°C and 32°C (AC21 and AC32) and inactive rats at 21°C and 32°C (IN21 and IN32). *P < 0.05, **P < 0.01, ***P < 0.001 *vs*. AC21; ^#^P < 0.05, ^##^P < 0.01, ^###^P < 0.001 *vs.* IN21; ^!!^P < 0.01, ^!!!^P < 0.001 IN21 *vs.* AC21. Data expressed as mean ± SEM.

AN is also characterized by a great loss of fat mass (38, 47, 48). Therefore, we decided to explore lipogenesis and lipolysis markers in the WAT of ABA rats. Active rats at 21°C had an extreme decrease in all examined lipogenic markers levels, such as acetyl-CoA carboxylate α (ACCα), fatty acid synthase (FAS), sterol regulatory element-binding protein 1 (SREBP1), peroxisome proliferator-activated receptor-gamma (PPARγ) and CCAAT/enhancer binding protein alpha and beta (C/EBPα and C/EBPβ), compared to inactive rats. Active rats at 32°C showed a marked recovery in the expression of these factors (**Figure 2A**). Increased housing temperature did not impact on gene expression levels in inactive rats, except for an increase in FAS and SREBP1 mRNA expression (**Figure 2A**). On the other hand, the levels of lipolysis markers, such as hormone-sensitive lipase (HSL), lipoprotein lipase (LPL), adrenergic receptor beta 1 (ADRβ1) and adrenergic receptor beta 3 (ADRβ3), but not beta 2 (ADRβ2), fibroblast growth factor 21 (FGF21) and carnitine palmitoyltransferase 1B (CPT1B), were reduced in active rats at 21°C, likely due to the massive loss of adiposity of these animals, while heat reversed this expression (**Figure 2A**).

AN patients have a reduction in lean mass and wasting syndrome, leading to cachexia (3, 49, 50). Therefore, we explored two cachexia markers in skeletal muscle, namely Atrogin-1 and Murf-1. Our data showed that rats housed at 21°C exhibited a markedly increased expression of cachectic markers relative to rats housed at 32°C, evident both in the inactive and the active cohort, possibly indicating muscle deteriorating (**Figure 2B**).

### ABA Reduces BAT UCP1 Levels But Increases the Browning of WAT

**Figure 3:**
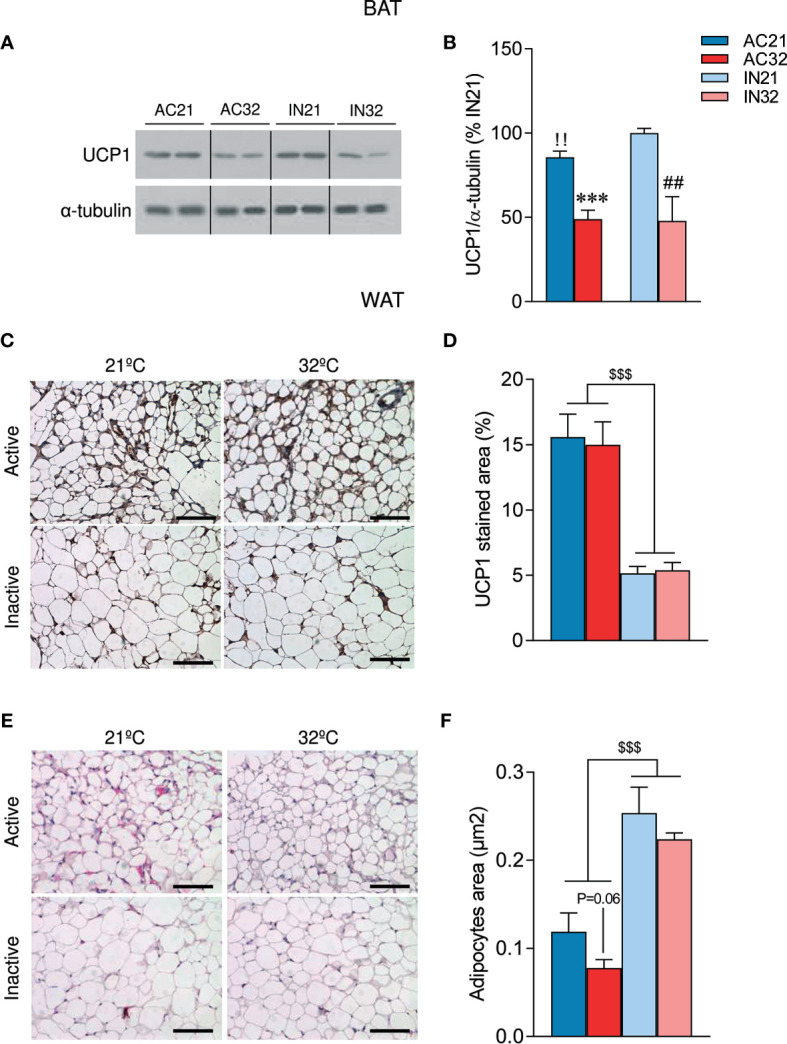
Effect of ABA and temperature on BAT and WAT browning. **(A, B)** Protein levels of UCP1 in the BAT (n = 7 rats/group) **(C, D)** UCP1 staining in WAT (n = 8-12 rats/group) **(E, F)** Adipocyte are in WAT (n = 8-12 rats/group) of active rats at 21°C and 32°C (AC21 and AC32) and inactive rats at 21°C and 32°C (IN21 and IN32). ***P < 0.001 *vs.* AC21; ^##^P < 0.01 *vs.* IN21; ^!!^P < 0.01 IN21 *vs.* AC21; ^$$$^P < 0.001 for simplification. Data expressed as mean ± SEM. The bands in gels from panel **(A)** have been spliced from the same original gels. Scale bar: 100 µm.

It is known that AN is associated with impaired thermogenesis (50). In fact, it has been reported that young women with AN exhibit reduced cold-activated BAT (50). Analysis of UCP1 expression in the BAT of our model showed decreased 21°C-induced UCP1 protein levels in AC rats (**Figures 3A, B**). As expected, increased environmental temperature decreased UCP1 expression in both active and inactive animals (**Figures 3A, B**).

Over the last years, accumulating evidence have demonstrated that activation of beige/brite (“*brown in white”*) adipocytes in the WAT, a process known as browning (51–53), is responsible for a significant increase in total energy expenditure (54). Notably, recent studies have also linked the browning of WAT to other wasting syndromes, such as cancer-induced cachexia (55, 56); however, to date, no data have linked AN to browning of WAT. Our histological analysis of WAT showed that ABA rats exhibited a *“brown-like”* multilocular pattern, associated with increased UCP1 immunostaining (**Figures 3C, D**) and decreased adipocyte area (**Figures 3E, F**). Importantly, the induction of browning was not affected by housing temperature (**Figures 3C–F**). Overall, these data indicate that ABA rats, besides hypophagia, also displayed increased browning of WAT, that was compatible with the elevated catabolic state.

### ABA Does Not Impact Either AMPK or ER Stress in the Hypothalamus

**Figure 4:**
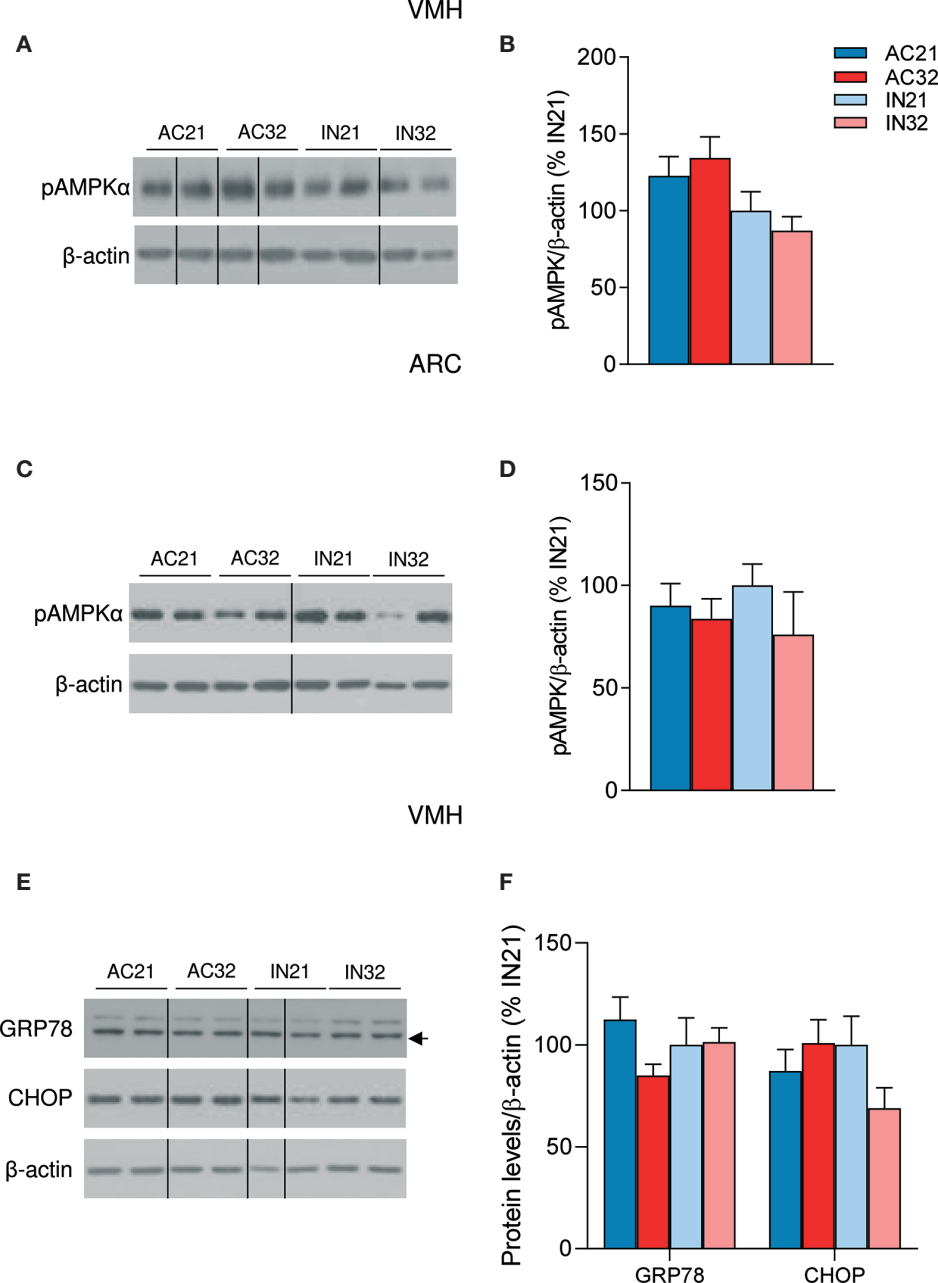
Effect of ABA and temperature on AMPK and ER stress in the VMH and ARC. **(A, B)** Protein levels of pAMPKα in the VMH (n = 7-10 rats/group) **(C, D)** Protein levels of pAMPKα in the ARC (n = 7 rats/group) **(E, F)** Protein levels of GRP78 and CHOP in the VMH (n = 7 rats/group) of active rats at 21°C and 32°C (AC21 and AC32) and inactive rats at 21°C and 32°C (IN21 and IN32). Data expressed as mean ± SEM. The bands in gels from panel **(A, C, E)** have been spliced from the same original gels.

Finally, we aimed to investigate if ABA might result in changes at the central level that could explain the catabolic state of this model. One of the principal regulators of energy balance at a central level is hypothalamic AMPK, an energy sensor that controls both sides of the energy balance equation: food intake and energy expenditure (20–22). Firstly, we investigated the effect of ABA and temperature on total hypothalamic extracts; our data did not show any significant impact of either ABA or temperature on the protein levels of the AMPK signaling pathway (**Supplementary Figure 1A**). Current data indicate that the effects of AMPK in the hypothalamus are nucleus-specific; thus while AMPK in the ARC is mainly involved in the regulation of feeding, AMPK in the VMH regulates BAT thermogenesis and browning of WAT (19–23, 32), Therefore, we performed further analysis of AMPK in ARC and VMH enriched protein lysates, which showed a non-significant tendency of phosphorylated AMPK (pAMPK) to be increased in the VMH of ABA rats, that might account for the decreased levels of BAT UCP1 protein levels observed in those animals (**Figures 4A, B**). No major effect of housing temperature was detected of pAMPK levels in the VMH (**Figures 4A, B**). Similar data were found when pAMPK was assayed in the ARC (**Figures 4C, D**). Overall, these results indicated that the impaired feeding and browning that characterized ABA model were unlikely associated to changes in AMPK signaling in these nuclei.

Finally, we investigated the effect of ABA on hypothalamic ER stress signaling, since recent data have linked this cellular response with the regulation of BAT thermogenesis and browning of WAT (24–26, 28). Our data did not show any major impact of either ABA or housing temperature on two key hypothalamic ER stress markers, namely GRP78 and CHOP (**Figures 4E, F**), excluding their association in the BAT and metabolic alterations of this model.

## Discussion

Here, we show that the catabolic state that characterizes ABA is associated with major changes in BAT thermogenesis and WAT browning. Notably, those changes are not related to modification in key central regulators of adipose tissue activity, namely hypothalamic AMPK and ER stress signaling.

AN is characterized by energy balance impairment as a result of decreased food intake and hyperactivity, leading to severe weight loss (1, 2). Different brain regions, such as reward-motivated learning or hippocampal structures, have been involved in the pathology of AN (1, 2, 8, 57). Here, we aimed to investigate whether the canonical hypothalamic (VMH)-AMPK-ER stress-SNS-BAT axis (21, 22) could be involved in the reduced feeding and the changes in BAT and WAT browning that characterize the ABA model in rats.

ABA is considered the best analogue animal model for AN (6). In addition, it is well-established that ABA-induced hyperactivity is an adaptative behavioral response to compensate for hypothermia (9). Our data are in line with previous studies reporting the beneficial effect of increased ambient temperature to 32°C on the recovery of rats subjected to the ABA model, even after 20% weight loss has occurred (10–13, 15, 58). Although our data confirm former evidence, the use of temperature recording by telemetry allows a constant monitoring of the body temperature throughout the experiment, which constitutes a big advantage when compared to previous reports. Food-restricted rats suffered hypothermia when given free access to a running wheel, as body temperature decreases over days. On the contrary, rats exposed to 32°C, both active and inactive, avoid hypothermia and their body temperature at the end of the experiment reached values higher than when meeting the weight loss criterion. These findings reinforce the hypothesis that hyperactivity is an adaptive response to compensate for the hypothermia derived from weight loss (9).

In mammals, the BAT is responsible for the adaptative thermogenesis which regulates body temperature when other mechanisms (i.e., heat conservation) are not enough to maintain homeothermy (59, 60). Food-restriction elicits reductions in energy expenditure through decreased BAT thermogenesis, as a strategy to save energy, although it leads to a hypothermic state (61–63). In ABA rats this response is exacerbated, entering in a vicious cycle situation that potentiates an overall catabolic state leading to wasting and cachexia. Notably, the increase in housing temperature reduced the expression of UCP1 in the BAT. That reduction in adaptive thermogenesis together with the reduction of hyperactivity would account for a better preservation of body mass (16) and the recovery of body weight of rats exposed to the ABA model. This is also demonstrated by an improved metabolic profile at the higher ambient temperature, exemplified by the reduction in the expression of cachectic markers in skeletal muscle and the increased WAT lipogenesis. Still, the recovery in body mass is not total, likely due to the maintained browning of WAT, which may account for a chronic increased energy expenditure (54), leading to a sustained basal catabolic state.

There are a huge amount of data linking hypothalamic AMPK and ER stress pathways in the hypothalamus, specifically in the VMH, with the regulation of thermogenesis in BAT, as well as the browning of WAT (19–26). This prompted us to investigate whether those hypothalamic molecular mediators could be associated to the BAT and WAT responses in the ABA model. Our analysis did not find major expression differences in the levels of pAMPK (the active isoform), GRP78 and CHOP either in the VMH and/or the ARC of ABA rats. In fact, this result is opposite to a recent report where it has been described that hypothalamic pAMPK levels are reduced in ABA mice (64). These discrepancies could be likely explained by the different species (rats *vs.* mice), but also by the nuclei-specific an analysis performed in our study, which is critical to understand AMPK and ER stress function in the hypothalamus (19–26) at the studied times. Moreover, timing could be also a factor, in this sense it is likely that at the final time point that we investigated, initial changes in hypothalamic AMPK and/or ER stress (maybe responsible for the BAT and browning changes observed) could not be detected. Further work will be needed to address the exact role of these molecular mechanisms in the pathology of AN.

In summary, our study shows a general description of the metabolic state of rats exposed to the ABA model and of those rats treated with heat. The results are consistent with the hypothesis that body temperature is an important parameter in ABA. The application of heat reverses not only the hyperactivity and weight loss of animals exposed to the ABA model, but also hypothermia, hypoleptinemia and loss of muscle mass. However, none of the changes observed are associated to changes in key hypothalamic pathways modulating energy metabolism, such as AMPK or ER stress (19–26) at the studied times. Hence, hypothermia in AN should be given more attention in future research to study the underlying brain mechanism involved in the warming effect and to explore new treatments.

## Data Availability Statement

The raw data supporting the conclusions of this article will be made available by the authors, without undue reservation.

## Ethics Statement

The animal study was reviewed and approved by The Ethics Committee on the use and care of animals of Santiago de Compostela University approved all described procedures (project license 15004/17/002). All experiments were carried out in accordance with Royal Decree 53/2013 of February 1, Law 32/2007 of November 7, and European Communities Council Directive 2010/63/UE of September 22, on the protection of animals used for experimental and other scientific purposes.

## Author Contributions

AF and ER-P performed the *in vivo* experiments, analytical methods and collected the data. AF, EG and ML designed the experiments and analyzed the data. AF, ER-P, RN, JF, CD, EG and ML interpreted and discussed the data. AF, EG and ML developed the hypothesis. AF and ML made the figures and wrote the manuscript. ML is the senior author and lead contact, secured funding, coordinated, and led the project. All authors contributed to the article and approved the submitted version.

## Funding

The research leading to these results has received funding from the Xunta de Galicia (RN: 2016-PG057; ML: 2016-PG068); Ministerio de Economía y Competitividad (MINECO) co-funded by the FEDER Program of EU (RN: RTI2018-099413-B-I00; CD: BFU2017-87721-P; ML: RTI2018-101840-B-I00); Atresmedia Corporación (RN and ML); Fundación BBVA (RN); “la Caixa” Foundation (ID 100010434), under the agreements LCF/PR/HR19/52160016 (RN) and LCF/PR/HR19/52160022 (ML); European Foundation for the Study of Diabetes (RN), ERC Synergy Grant-2019-WATCH- 810331 (RN) and Western Norway Regional Health Authority (Helse Vest RHF) (JF). AF received a fellowship from Xunta de Galicia (Plan I2C-2014). The CiMUS is supported by the Xunta de Galicia (2016-2019, ED431G/05). CIBER de Fisiopatología de la Obesidad y Nutrición is an initiative of ISCIII. The funders had no role in study design, data collection and analysis, decision to publish, or preparation of the manuscript.

## Conflict of Interest

The authors declare that the research was conducted in the absence of any commercial or financial relationships that could be construed as a potential conflict of interest.
