# Supplementary material for: Activity-Based Anorexia Induces Browning of Adipose Tissue Independent of Hypothalamic AMPK
Source: Front Endocrinol (Lausanne). 2021 Jun 2;12:669980. doi: 10.3389/fendo.2021.669980 (PMC8206787; doi:10.3389/fendo.2021.669980)
Supplement: Supplementary file 1 [file DataSheet_1.pdf]

Supplemental Table 1. Primers and probes for real-time PCR (TaqMan) analysis

Gen	GenBank	Function	Sequence
ACCα	NM_022193	Fw	5'-TGGGCGGGATGGTCTCTTT-3'
		Rv	5'-AGTCGCAGAAGCAGCCCATT-3'
		Pb	FAM-5´-ACCTTTGAAGATTTCGTCAGGATCTTTGATGA-3'-TAMRA
		Fw	5´-TCTACGTGCCCCTGTGCAT-3
ADRβ1	NM_012701	Rv	5´-GCTGTCGATCTTCTTCACCTGTTT-3'
		Pb	FAM-5´-TGTACCTCCGGGTGTTCCGCGA-3´-TAMRA
		Fw	5´-CTTCCAGGTGGCCAAAAG-3´
ADRβ2	NM_012492	Rv	5´-GCTGAGGTTTTGGGCATGAA-3´
		Pb	FAM-5´-TGCAGAAGATAGACAAATCCGAGGGCAG-3´-TAMRA
		Fw	5´-CCTTCCCAGCTAGCCCTGTT-3´
ADRβ3	NM_013108	Rv	5´-TGCTAGATCTCCATGGTCCTTCA-3´
		Pb	FAM-5´-AACTCACCGCTCAACAGGTTTGATGGC-3´-TAMRA
ATROGIN-1	NM_133521.1	Assay ID	ThermoFisher Taqman Gene Expression Assays Assay ID Rn01504258_m1
C/EBPα	NM_001287579.1	Fw	5'-GTGCGTAAGAGCCGAGATAA-3'
		Rv	5'-TGGTCAACTCCAACACCTTCTG-3'
		Pb	FAM-5'-AAACAGCGCAACGTGGAGACGCA-3'
C/EBPβ	NM_024125.5	Fw	5'CAGAGTAGCCCCTTGTCTTTTGG-3'
		Rv	5'-GGATTGCAGGTCAGACACAGAA-3'
		Pb	FAM-5'-TGAGGATAGCCAGGTCAAAGCCCA-3'
CPT1b	NM_013200.1	Assay ID	Applied Biosystems TaqMan Gene Expression Assays Assay ID Rn00566242_m1
FAS	NM_017332	Fw	5'-GACATTTCATCAGGCCACC-3'
		Rv	5'-CCTCTAGCAGCCGCACCTC-3'
		Pb	FAM-5'-CTGCCCAGGACAGGAACCG-3'-TAMRA
		Fw	5´-CAC TCT GGT ACC CAG GAC CAA TAA-3´
FGF21	NM_130752	Rv	5´-CCC TCA ACT TTT CTC TGC CTA GGT-3´
		Pb	5´-ACA AGA GTA GAG GTG GTG GGC AGA ATG CC-3´
HPRT	NM_012859	Fw	5'-AGCCGACCGGTTCTGTCAT-3'
		Rv	5'-GGTCATAACCTGGTTCATCATCAC-3'
		Pb	FAM-5'-CGACCCTCAGTCCCAGCGTCGTGAT-3'-TAMRA
HSL	NM_012859	Fw	5'-CCAAGTGTGTGAGCGCCTATT-3'
		Rv	5'-TCACGCCCAATGCCTTCT-3'
		Pb	FAM-5'-AGGGACAGAGACGGAGGACCATTTTGACTC-3'-TAMRA
LPL	NM_012598	Fw	5'-CTGAAAGTGAGAACATTCCCTTCA-3'

		Rv	5’-CCGTGTAAATCAAGAAGGAGTAGGTT-3’
		Pb	FAM-5’-CCTGCCGGAGGTCGCCACAAATA-3’-TAMRA
MURF-1	NM_080903.1	Assay ID	ThermoFisher Taqman Gene Expression Assays Assay ID Rn01639111_m1
PPARγ	NM_013124	Fw	5'-TGGGCCAGAATGGCATCTC-3'
		Rv	5'-CTGATGCACTGCCTATGAGCACTTCACA-3'
		Pb	FAM-5'-CTAACTCCCAGAAAAGCAAGCAA-3'-TAMRA
SREBP1	NM_011480	Fw	5´-CTCATCAACAACCAAGACAG-3´
		Rv	5´- TGTTGCCATGGAGATAGCATCTC-3´
		Pb	FAM-5’-ACCGGCAGCCTTTTTCAAAGGGTTTG-3’-TAMRA

A

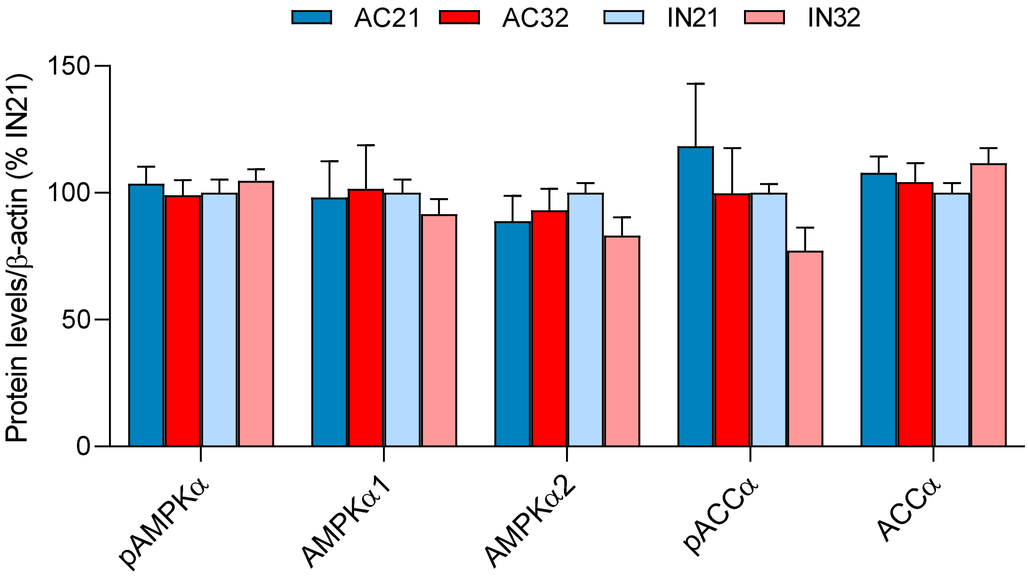

Supplemental Figure 1. Effect of ABA and temperature on hypothalamic AMPK

(A) Protein levels of AMPK pathway in the hypothalamus (n= 7 rats/group) of active rats at 21ºC and 32 ºC (AC21 and AC32) and inactive rats at 21ºC and 32 ºC (IN21 and IN32). Data expressed as mean±SEM.
